# Lived experiences of Palestinian patients with COVID-19: a multi-center descriptive phenomenological study of recovery journey

**DOI:** 10.1186/s12889-022-12868-9

**Published:** 2022-03-09

**Authors:** Aidah Alkaissi, Fadi Zaben, Mohammad Abu-Rajab, Mahdia Alkony

**Affiliations:** Nursing and Midwifery Department, Faculty of Medicine & Health Sciences- An-Najah National University, Nablus, Palestine

**Keywords:** COVID-19, SARS-CoV-2, Experiences, Quarantine, Coping, Stigma, Palestine

## Abstract

**Background:**

Exploring lived experiences of recovered COVID-19 patients might have scientific, social, and policy relevance that can apply to the healthcare infrastructure. This multi-center descriptive phenomenological study was conducted to explore lived experiences of Palestinian patients who recovered from COVID-19.

**Methods:**

This was a descriptive phenomenological study. A purposive sampling technique was used to recruit the study participants. Semi-structured qualitative interviews were conducted with patients who recovered from COVID-19 (*n = 20*). The interviews were transcribed verbatim. The transcripts were analyzed using Colaizzi’s phenomenological approach which consisted of the following steps: 1) familiarization, 2) identification of significant statements, 3) formulation of the meanings, 4) clustering the themes, 5) development of an exhaustive description of the phenomenon, 6) production of the fundamental structure, and 7) verification of the fundamental structure.

**Results:**

Semi-structured interviews were conducted with 14 male and 6 female patients who recovered from COVID-19. The total duration of the interview time was 998 min (16.6 h). The qualitative data collected during the interviews were categorized into 5 major themes and 16 subthemes that exhaustively described the phenomenon. The major themes were relevant to: 1) emotions after learning about the infection, 2) experiencing social exclusion and stigma, 3) the experienced symptoms, 4) supportive treatments, herbs, rituals, and social support, 5) and life after recovery.

**Conclusion:**

The interviewees recounted experiencing negative emotions, social exclusion, and stigma because of their infection. It may be important for mental health promotion to be an integral part of the care plan for patients with COVID-19. More studies are still needed to investigate if introducing mental healthcare providers to the care team of patients with COVID-19 can improve the experiences of the patients.

**Supplementary Information:**

The online version contains supplementary material available at 10.1186/s12889-022-12868-9.

## Background

In 2019, a new coronavirus (COVID-19) was identified as the causative agent of an outbreak of respiratory disease that originated in Wuhan, China. Afterwards, the virus spread to several other countries around the world and the World Health Organization (WHO) officially declared this global outbreak a pandemic [1]. In Palestine, the first case tested positive for COVID-19 on March 5, 2020. As of November 17, 2021, there were 457,731 confirmed cases of COVID-19 including 4767 deaths as per the statistics of the Palestinian Ministry of Health [2].

The virus is transmitted through direct and indirect contact with infected persons and contaminated objects [3, 4]. Person-to-person transmissions occur mainly during close contact with an infected individual, contact with droplets produced by coughing, and/or contact with droplets produced while sneezing and talking [5]. Indirect transmission occurs by touching contaminated surfaces or objects and then touching the face. Although the virus is more contagious in the first days after the onset of symptoms, asymptomatic patients can also spread the disease [6, 7].

The full spectrum of COVID-19 infection ranges from subclinical self-limiting respiratory disease to severe progressive pneumonia with multiple organ failures and death [8]. According to studies and reports, more than 80% of patients remained asymptomatic and 15% of the patients developed mild symptoms [6, 9]. Fever, cough, dyspnea, myalgia, and fatigue were the most commonly reported symptoms of the disease [8].

Exploring lived experiences of healthcare providers during the ongoing pandemic has received considerable attention [10–13]. As the pandemic is still unfolding, exploring the lived experiences of the patients would also be interesting. In recent studies, patients who recovered from COVID-19 recounted distressing mental health problems that they experienced during isolation periods [10, 11]. Other studies have revealed how COVID-19 patients managed their emotions within the isolation settings [14]. In a recent descriptive phenomenological study, Moradi et al. uncovered psychological reactions of COVID-19 patients to stress caused by the disease [15]. Psychological maladaptation and self-moderating effect of stress were identified as the two major themes. Liu and Liu conducted a phenomenological study among hospitalized COVID-19 patients who were involved in family cluster transmission [10]. The themes that emerged from the qualitative data collected in the study uncovered the widespread prejudice, sadness, uncertainty, and fear of death experienced by the patients.

It has been argued that awareness of the lived experiences of recovered COVID-19 patients could be informative to decision makers who might be interested in designing interventions to improve the physical environment and delivery of care/support to reduce the negative consequences that could be brought about by the isolation period [16]. Considering resurgence of the pandemic in different geographical regions around the world, there is still an increasing need to shed more light into the lived experiences of the patients who recovered from COVID-19, understand the effects of the isolation period on their mental health, explore how they managed their symptoms, and learn from their experiences [10, 11, 17–19]. This might help decision makers improve isolation environments of future patients.

In Palestine, patients with confirmed COVID-19 infection are destined for isolation either in a healthcare center designated for COVID-19 patients or at their homes. Little is known on the lived experiences of patients who recovered from COVID-19 in Palestine. This phenomenological study was conducted to explore lived experiences of Palestinian patients who recovered from COVID-19, understand the effects of the isolation period on their mental health, explore how they managed their symptoms, and learn from their experiences. The study shed light on the real-world experiences of the patients during the isolation period. Findings of this study might have scientific, social, and policy relevance that apply to the healthcare infrastructure in Palestine and other developing countries.

## Methods

### Study design

This was a descriptive phenomenological study. In descriptive phenomenological studies, human experiences are often accurately described through qualitative data that are collected in interviews [20, 21]. It has been argued that descriptive phenomenology is the ideal approach to explore the universal aspects of a new phenomenon that has never been conceptualized before [22]. Infection with COVID-19 is a new phenomenon that Palestinian patients have experienced for the first time. In nursing research, descriptive phenomenological approaches are commonly used to describe lived human experiences and explore perspectives of the interviewees with regard to a phenomenon of interest [23, 24]. In this study, the phenomenon of interest was lived experiences of Palestinian patients while being isolated as a result of infection with COVID-19.

During the interviews, lived experiences of the participants were explored after recovering from the infection. In this qualitative phenomenological study, a holistic approach was followed to understand how the experiences of the participants were contextually formed, influenced, and maintained during both infection and isolation periods. This study was conducted and reported in adherence to the consolidated criteria for reporting qualitative research (COREQ) [25]. Adherence to the checklist is provided in Supplementary Table S1.

### Participants

A purposive sampling technique was employed to select the study participants. The participants in this study were Palestinian patients who tested positive for COVID-19 infection and were isolated either in their homes or in healthcare centers designated for patients with COVID-19. As the researchers in this study were academician nurses, the study participants were identified and invited to participate in this study through personal contacts in the field and community. The inclusion criteria were as follows: 1) being at least 18 years old, 2) tested positive for COVID-19 using the standard polymerase chain reaction (PCR) test, 3) completed an isolation period of at least 2 weeks, and 4) providing an informed consent to participate in the study. Patients who reported active or past history of psychiatric disorders and those with severe cognitive impairments were excluded from this study. The study participants were informed that this study was being conducted to explore their lived experiences during the isolation period, understand the effects of the isolation period on their mental health, explore how they managed their symptoms, and learn from their experiences.

### Sample size

The sample size for this study was informed by previous descriptive phenomenological studies that were conducted among COVID-19 patients [10, 26]. In this study, the thematic saturation point was used as an adaptive approach to determine the sample size [27]. We assumed a priori that at least 800 min of interview time would be sufficient to generate qualitative data that would allow achieving thematic saturation [28]. Assuming an average interview time of 40 min, at least 20 interviews would be needed in this study. In this study, no further interviews were conducted when thematic saturation was achieved.

### Development and pilot testing of the interview guide

The probing questions were developed after a literature review and consultations with the experts in the field [10–12, 26]. To ensure face validity, the questions in the interview guide were rated for suitability by 4 academicians who were also practicing nurses [29]. Discrepancies were discussed and resolved through consensus. Because the study participants were native Arabic speakers, the questions in the interview guide were translated from English to Arabic. To ensure accuracy, forward and backward translations were carried out by bilingual independent translators. To avoid any potential bias, the back-translators were not aware of the intended concepts the questions were designed to probe [30]. Discrepancies were discussed and resolved through consensus. Before the interviews were conducted, the interview guide was pilot tested among 2 COVID-19 patients to ensure using relevant terminologies and generation of sequential data. The questions in the interview guide were related to the symptoms, diagnosis, time in isolation, and physical, psychological, and social health of the participants. The interview guide is provided in Supplementary Table S2.

### Data collection

As the study was conducted during the ongoing pandemic, the qualitative data were collected through semi-structured interviews conducted through a proprietary videotelephony software program (Zoom, San Jose, California) in full compliance with the physical distancing instructions. The Zoom meeting was private and password protected to ensure privacy and comfort of the participants.

After establishing contact, the scope and objectives of the study were explained to the potential participants. A verbal consent was obtained from each participant before the interview. The interviews were conducted by 4 researchers (2 male and 2 female) who were employed by the main teaching university in Palestine at the time of the study. Of the researchers, 3 had Master of Science degrees (MSc in nursing) and 1 had a doctorate (PhD in nursing). The researchers were trained as nurses and had experience in interviewing patients to obtain health information. To ensure data saturation within the theoretical background and framework of analysis, each interview lasted for at least 40 min.

The study participants were interviewed while they were at home. Before each interview, sociodemographic characteristics of the participants like age, gender, place of residence, marital status, number of children, number of family members, educational level, smoking status, occupation, and monthly income were collected. We also collected information on the presence of cardiovascular, digestive, endocrine, pulmonary, and other diseases.

The interviews started with an open-ended question on how the patient felt about having contracted COVID-19 and recovered from the disease. The participants were asked to recount their lived experiences from the time they received their diagnosis through recovery (Supplementary Table S2). Whenever needed, prompts were used to clarify, examine, and/or unpack some answers/experiences. The interviews were conducted during the month of February 2021.

Planning considered providing adequate psychological interventions to participants who might exhibit emotional distress during the interviews to prevent potential psychological harm. To ensure authenticity of the data collected during the interviews, the interviewers actively listened to the participants, clarified information whenever needed, and unconditionally accepted the information/experiences described by the participants. As the interviews were recorded, field notes were seldom taken.

### Data analysis

All interviews were recorded and transcribed verbatim. The qualitative data collected during the interviews were translated from Arabic into English and back translated into Arabic again to ensure accuracy. The transcripts were independently analyzed using Colaizzi’s phenomenological approach by the 4 researchers [31]. This approach consisted of the following 7 steps: 1) familiarization: each researcher read the transcripts 3–5 times to understand the meaning of the qualitative data, 2) identification of significant statements: the researchers identified all statements that could be of relevance to the phenomenon, 3) formulation of the meanings: the researchers identified all meanings that could be relevant to the phenomenon that raised after carefully considering the significance of the statements, 4) clustering the themes: the researchers clustered the identified meanings into themes, 5) development of an exhaustive description: the researchers detailed an exhaustive description of the phenomenon incorporating the themes clustered in step (4), 6) production of the fundamental structure: the researchers condensed the exhaustive description into a short and dense statements that captured the essential aspects of the phenomenon, and 7) verification of the fundamental structure: the researchers retuned the fundamental statements to the participants to verify if it accurately captured their experience [31, 32].

To ensure trustworthiness and credibility, bracketing was used throughout this study to set personal knowledge, experience, and expectations of the researchers aside and avoid preconception bias [33]. Audit trails of analytical decisions were used to ensure dependability. To ensure the accuracy and strengthen the qualitative thematic analysis, a number of established techniques were used. These techniques included close and repeated reading of transcripts, regular discussion about new results among the research team members using disagreements between researchers to request a search for confirmation or rejection of data, and triangulation of results from individual interviews with the participants [34]. The transcripts were reviewed again to establish relationships between the themes, subthemes, and patterns identified. The qualitative data collected in this study were grouped into themes, subthemes, and patterns by the 4 researchers independently. Theme contents were compared and matched. Discrepancies were discussed and resolved through consensus among the research group.

### Ethics approval and consent to participate

The study was conducted in adherence to the international ethical considerations in the Declaration of Helsinki [35]. The study received approval from the Institutional Review Board (IRB) of An-Najah National University. Because this was a descriptive phenomenological study that was conducted during the on-going COVID-19 pandemic while a stay-at-home order was effective, a verbal consent was required from the participants before they were interviewed. The IRB of An-Najah National University approved this verbal consent. Confidentiality of the participants was maintained and no information leading to the identification of the participants was disclosed. The recorded materials were kept in a password protected safe.

## Results

In this study, semi-structured interviews (*n = 20*) were conducted with 14 male and 6 female patients who recovered from COVID-19. The total duration of the interview time was 998 min (16.6 h). The age of the participants ranged from 25 to 50 years. The participants were recruited from different geographical regions of Palestine. The participants were diverse in terms of marital status, number of children, family size, educational level, smoking status, occupation, income, and having other comorbidities. The detailed sociodemographic and health characteristics of the participants are shown in Table 1.

**Table 1:** Sociodemographic and health details of the interviewees

#	Age (years)	Gender	Place of residence	Marital status	Number of offspring	Number of family members	Education	Smoking	Occupation	Previous history	Monthly income (NIS)
P1	25	Male	Tulkarm	Married	0	2	University degree	Yes	Nurse	Gastro esophageal reflex and Asthma	≥ 3000
P2	48	Male	Nablus	Married	3	5	School	No	Police officer	High blood pressure, hyperlipidemia and hypercholesterolemia	≥ 3000
P3	27	Male	Tulkarm	Single	0	0	University degree	No	Nurse	ND	≥ 3000
P4	49	Male	Tulkarm	Married	4	6	University degree	No	Businessman	ND	≥ 3000
P5	50	Female	Tulkarm	Married	5	7	University degree	No	School teacher	Hypertension	≥ 3000
P6	48	Female	Nablus	Married	8	10	University degree	No	Midwife	ND	≥ 3000
P7	29	Male	Nablus	Single	0	1	University degree	No	Secretary	ND	2000–3000
P8	38	Male	Nablus	Married	2	4	School	Yes	Carpenter	ND	≥ 3000
P9	35	Male	Bethlehem	Married	3	5	University degree	No	Nurse	ND	≥ 3000
P10	28	Male	Nablus	Single	0	5	University degree	No	IT	ND	≥ 3000
P11	26	Female	Ramallah	Single	0	11	University degree	No	ND	ND	ND
P12	26	Male	Jenin	Single	0	5	University degree	No	Nurse	ND	≥ 3000
P13	31	Male	Hebron	Married	2	6	University degree	Yes	Nurse	ND	≥ 3000
P14	50	Female	Nablus	Married	5	7	University degree	No	School teacher	ND	≥ 3000
P15	25	Male	Nablus	Single	0	0	University degree	No	Police officer	ND	≥ 3000
P16	30	Male	Nablus	Single	0	5	University degree	Yes	School teacher	ND	2500
P17	26	Male	Nablus	Single	0	5	University degree	No	IT	ND	≥ 3000
P18	55	Female	Nablus	Married	3	4	University degree	No	School teacher	ND	≥ 3000
P19	49	Female	Ramallah	Married	4	6	University degree	Yes	School teacher	ND	≥ 3000
P20	45	Male	Nablus	Married	3	5	University degree	No	School teacher	ND	≥ 3000

*ND* not disclosed, *NIS* New Israeli Shekel, *IT* information technology

The qualitative data collected during the interviews were categorized into 5 major themes and 16 subthemes that exhaustively described the phenomenon. The major themes were relevant to: 1) emotions after learning about the infection, 2) experiencing social exclusion and stigma, 3) the experienced symptoms, 4) supportive treatments, herbs, rituals, and social support, 5) and life after recovery. Relevant quotations were provided under each theme and subtheme. Additional quotations are provided in the supplementary materials.

### Major theme 1: emotions after learning about the infection

#### Subtheme 1: shock/disbelief (feeling awful, anxious, and shocked)

All of the participants stated that they felt shocked to learn that they tested positive for the virus. Receiving the diagnosis was met with anxiety and shock. One of the participants shared:

*“It felt a bit awful that I actually got infected by coronavirus... when I tested positive, I couldn't believe that I had actually contracted the virus” (P2).*

#### Subtheme 2: denial (feeling doubtful)

All the participants stated that they felt doubtful about the positive result of the test as they experienced either mild or non-specific symptoms. The symptoms typically included sore throat, low-grade fever, muscle ache, nausea, and vomiting. This doubt was substantiated by either misdiagnosis as either common cold or seasonal flu when they visited their physicians or being adherent to hygiene practices, wearing masks, and gloves. One of the participants stated:

*“I had symptoms of pain in the throat and a high temperature. The doctor told me that it was not corona and prescribed me an antibiotic and antipyretic. On the third day, I lost the sense of smell and the sense of taste. I did a PCR and I tested positive…” (P15)*

#### Subtheme 3: feeling angry/frustrated and imprisoned

The participants reported experiencing negative emotions like feeling imprisoned, anger and frustration due to the fact that patients with COVID-19 would be subjected to an isolation period. One of the participants shared:

“*It was a nightmare and in fact every time I slept, I had nightmares. It was the worst period of my whole life”* (P6)

#### Subtheme 4: bargaining/spirituality to cope with adversity

Some of the participants stated that they have turned to spirituality and sought help from God to pass this period of adversity. One of the participants expressed feeling closer to God through prayers and reading the Holy Quran. One of the participants expressed:

*“I did not lose hope in God… praise be to God always and forever”. (P13)*

#### Subtheme 5: feeling guilty

All of the participants expressed concerns that they could spread the infection to others, especially, their family members. Some of the participants expressed feeling embarrassed and guilty that they had passed the virus to their family members. One participant expressed:

*“I was the first person to contract the virus and passed it to my wife and children. I felt guilty” (P5)*

#### Subtheme 6: depression (feeling depressed and lonely)

The participants reported that they had depressed mood, felt lonely, boredom, had nightmares, and feared death during the period of isolation. One of the participants shared:

*“I suffered depression and I was feeling lonely” (P1)*

#### Subtheme 7: acceptance and hope (being positive)

The participants stated that they have accepted the fact that they have contracted the virus and tried to be positive. One of the participants stated:

*“Despite that I felt bored during the isolation period. I was willing to stay way to protect others from contracting the virus” (P3)*

### Major theme 2: experiencing social exclusion and stigma

Participants reported experiencing social exclusion and stigma in their communities. Some of the participants reported having to take longer walking routes to their homes as they were not allowed by the community members to walk in the main streets. One of the participants expressed concerned that in the future they and their family members would be socially excluded.

#### Subtheme 1: social exclusion and stigma while living with the infection

One of the participants recounted that the neighbors burned the trash can in front of their home as the ambulance came to transfer a patient to the hospital. One participant shared:

*“Me, my wife, and my four children tested positive for corona virus. The neighbors placed a label on our house that read “corona house”. We were isolated and bullied. I will never forget what has happened to us during that period. God forgive them [the neighbors] for what they did to us” (P16)*

#### Subtheme 2: social exclusion and stigma after recovery

The participants reported that social exclusion and stigma continued after recovery. The participants stated that even after testing negative for the virus people avoided any contact with. One participant shared:

*“When I returned back to my job, I noticed that my colleagues avoided contact with me or with anything that belonged to me. This made me feel that I was an outcast” (P6)*

### Major theme 3: the experienced symptoms

The symptoms experienced by the participants were reported as dry or purulent productive cough, fever, shortness of breath, loss of smell and taste, cold like symptoms, symptoms similar to seasonal flu, dry throat, thirst, nausea, vomiting, loss of appetite, muscle ache, fatigue like a rag, intolerable headaches, red eyes, blurred vision, heart palpitation, sweating at night, dizziness, feeling pressure in the ears, insomnia, have frequent night time awakenings, and feeling restless. In this study, all participants experienced respiratory symptoms. The participants shared:

*“I had persistent dry cough, fever, and shortness of breath …… I suffered severe myalgia and could not lift my arm… it was even hard for me to get out of bed...I had body aches, sore throat, and I loss the senses of smell and taste” (P17)*

### Major theme 4: supportive treatments, herbs, rituals, and social support

#### Subtheme 1: supportive treatments and herbs

In absence of preventive and therapeutic agents for COVID-19, the participants turned to herbal medicines and rituals. The participants reported using bronchodilators like salbutamol, anticholinergics like ipratropium, corticosteroids, analgesics like paracetamol, and antibiotics like azithromycin. The participants also reported turning to herbs. All participants reported using black seed (*Nigella sativa*), honey, boiled dry flowers, anise grain, chamomile, curcumin, lemon, and ginger to relieve their cough. The participants also reported to turn to healthy diet, fruits, and vegetables to boost their immunity.

The participants shared:

*“I used to grind black seed (Nigella sativa) with honey, boiled dry flowers, anise grain, chamomile, curcumin, lemon, and ginger to relieve my cough” (P4)*

#### Subtheme 2: rituals

The participants reported using posture (i.e., particular body positions), rituals, and other techniques to relief their symptoms. One of the participants shared:

*“I practiced prayer-mode prostration. This position helped relief my dyspnea” (P5)*

#### Subtheme 3: social support

The participants stressed the importance of receiving social support while experiencing an adversity. This made them feel loved, improved their mood, and helped them during the isolation period. The participants shared:

*“My fiancée used to come and talk to me from behind the window panes. This comforted me a lot” (P3)*

### Major theme 5: life after recovery

#### Subtheme 1: feeling relieved

Some of the participants expressed feeling relieved and were excited to go out again and see people after their recovery and completing their isolation period. One of the participants expressed:

*“I feel excited that I would go back and meet people, see the street, and go back to my work ...” (P12)*

#### Subtheme 2: adherence to preventive measures and adopting a healthy lifestyle

The participants reported adopting a healthy lifestyle to improve their immunity. The participants stated that experiencing infection with COVID-19 led them to change their behaviors and adopt a healthy lifestyle to improve their immunity. The participants reported that they would strictly adhere to preventive measures including frequent hand washing, wearing masks, gloves, practicing physical distancing, avoiding/minimizing physical contact, covering the mouth and nose while coughing, and avoiding contamination of the face with unwashed hands. One of the participants shared:

*“One should follow the instructions of health authorities and self-isolated. You have to protect your colleagues, friends, family, and yourself” (P1)*

#### Subtheme 3: fear of re-infection

Some participants expressed fear of re-infection. One participant shared:

*“Indeed, I am worried that I might contract the disease again. I do not know if my body has built antibodies or not. I am not sure if the disease has damaged some organs in my body. I am worried about my fertility” (P1)*

## Discussion

In this phenomenological study, patients who recovered from COVID-19 recounted their lived experiences from the moment they learned about their infection to the aftermaths this experience. The recovered patients who were interviewed in this study recounted experiencing negative emotions, social exclusion and stigma, and feared re-infection. Additionally, the interviewees recounted what they did to manage their symptoms, boost their immunity, and lift their spirits. To the best of our knowledge, this is the first study to explore lived experiences of Palestinian patients who recovered from COVID-19.

The emotions experienced by the patients after learning about their infection were consistent with the model introduced by Elisabeth Kübler-Ross [36, 37]. In this model, people cope with loss/bereavement/life-threatening incident by shock/disbelief, denial, anger, bargaining, guilt, depression, and finally acceptance/hope. It is important to note that these stages are neither universal nor necessarily linear [38]. Additionally, findings of this study were consistent with those reported by Wang et al. on the high prevalence of anxiety and depression among patients who contracted COVID-19 [39]. Moreover, patients who recovered from COVID-19 reported feeling depressed, overwhelmed, and had traumatic memory [10–12, 40–45]. Probably, these negative emotions were augmented by the misconceptions and lack of understanding of the nature of the disease, the environment in the isolation settings, and fear of transmitting the virus to family. Our findings might indicate that psychological support and direct communication might be needed to reduce the deleterious consequences of these negative emotions on the mental health of patients who recovered from COVID-19 [41, 42]. Mental health and psychological support specialists should become aware that anxiety, insomnia, shame, rage, resentment, and internalized stigma affect patients who recovered from COVID-19 and may need to be addressed [11, 41, 42]. Recently, the WHO recommended providing psychological support to the general public during the ongoing pandemic [11, 46, 47]. Because addressing mental health issues and providing psychological support are not part of the current treatment protocols of COVID-19, it was suggested that hospitals/healthcare centers to which COVID-19 patients are admitted need to offer mental health/psychological support and mental healthcare providers should be involved in screening patients with COVID-19 [40, 48–50].

As isolation is unavoidable in highly contagious diseases like COVID-19, findings of this study indicated the need to improve the isolation environments and communication channels between the patients and their friends and family [51]. Access to the internet, social media, and other telecommunication modalities could be helpful in improving communication between the patients and their beloved ones. Additionally, the physical features of the isolation settings may significantly impact perceptions of the patients and their experiences [52, 53]. It has been argued that sufficient space to walk around, large windows with a view of the surroundings/nature around the isolation place might help reduce the negative psychological consequences of the isolation period [53]. In the current study, some of the participants reported that they distracted themselves through watching TV, prayers to God, and reading the Holy Quran. The participants in this study stressed the importance of religion and reported finding new insights into their faith during the isolation period. In this study, most of the participants believed that stronger faith would protect them from the negative consequences of the disease. Previous studies have reported that religious beliefs shaped patients’ feelings as receiving reward from God for their spiritual experiences during isolation [54].

The physical symptoms reported by the participants in this study were consistent with those reported in previous studies conducted elsewhere [39, 55, 56]. Respiratory symptoms were commonly reported among patients with COVID-19 [57, 58]. Additionally, fever and dry cough were also reported as common symptoms. Dyspnea, nausea, vomiting, diarrhea, and abdominal pain were also reported [55, 58, 59]. Huang et al. (2020) reported that fever is the most common manifestation reported by patients (98%), followed by cough (76%), myalgia or fatigue (44%), sputum production (28%) and headache (8%) [9]. It is noteworthy mentioning that symptoms and their severity might can vary significantly among COVID-19 patients [39, 60].

Findings of this study indicated that the participants experienced social stigma and exclusion, consistent with previous studies conducted elsewhere [44, 45]. Social exclusion and stigma were previously shown to result in negative self-perception and lower rates of disclosure of infection [61]. This might jeopardize the health of the patients and the society at large. Previous studies have shown that stigmatized individuals can be subjects to social exclusion, physical violence, and denial of housing and care [62, 63]. In previous outbreaks of infectious diseases, stigma was also reported as a major issue and isolated patients were subjected to rejection from people in their local neighborhoods [64]. Survivors also reported differential treatment by others, avoidance, and withdrawal of social invitations [65–67]. The severity of stigmatization was associated with the length of the isolation period [68, 69]. Taken together, findings of this study may indicate a need for interventions to reduce stigma and social exclusion of patients who contracted COVID-19. This can encourage disclosure of infections and helps protect others from contracting the disease [67].

At the time of the study, there were no approved drugs to cure COVID-19. Therefore, the participants in this study used herbal medicines in a trial to improve their immunity and alleviate the symptoms of the disease. Findings of this study were consistent with previous studies in which patients tried herbal medicines to alleviate symptoms of viral infections [70–72]. Some herbal medicines are known to contain certain active compounds that exhibit antiviral, antimicrobial, anti-inflammatory, antioxidant, and immunostimulatory activities [73]. The herbal medicines reported in this study includes those derived from echinacea, quinine and curcumin [73]. Probably, herbal medicines can help modulate the immune responses and help alleviate the symptoms associated with COVID-19. In absence of solid scientific basis, herbal medicines are extensively offered in the markets claiming to cure certain diseases for which modern drugs are not available/approved. During the current pandemic, many herbal medicines are being sold with a claim to cure COVID-19. Rigorously designed clinical trials are still needed to investigate if such herbal medicines can be helpful in the treatment of COVID-19.

### Limitations of the study

There are a number of limitations that should be acknowledged when interpreting the findings of this study. First, participants experienced mild to moderate clinical symptoms and none of them was intubated. This can limit the findings described in this study to similar cases and hampers transferability of the findings to the entire population of COVID-19 patients. Therefore, future studies should include patients who experienced more severe clinical symptoms including those who were admitted to the intensive care units and those who were intubated. Second, the participants were recruited using a purposive sampling technique. This nonprobability sampling technique is inherently biased compared to other probability sampling techniques. Third, the patients were interviewed through a videotelephony software program, which may have influenced the quality and content of the data collected.

## Conclusion

Findings of this qualitative study described lived experiences of patients who contracted and recovered from COVID-19. The interviewees recounted experiencing negative emotions, social exclusion, and stigma because of their infection. It may be important for mental health promotion to be an integral part of the care plan for patients with COVID-19. More studies are still needed to investigate if introducing mental healthcare providers to the care team of patients with COVID-19 can improve the experiences of the patients.

## Supplementary Information

**Additional file 1.**

## Data Availability

All data relevant to this work are included within the manuscript or in the additional file as supplementary materials.

## Abbreviations

COVID-19: Coronavirus disease of 2019
COREQ: Consolidated criteria for reporting qualitative research
IRB: Institutional Review Board
NIS: New Israeli Shekel
SARS: Severe Acute Respiratory Syndrome
WHO: World Health Organization
