# Supplementary material for: Lived experiences of Palestinian patients with COVID-19: a multi-center descriptive phenomenological study of recovery journey
Source: BMC Public Health. 2022 Mar 9;22:470. doi: 10.1186/s12889-022-12868-9 (PMC8905007; doi:10.1186/s12889-022-12868-9)
Supplement: Supplementary file 1 — Additional file 1. [file 12889_2022_12868_MOESM1_ESM.docx]

**Supplementary materials for**

**Lived experiences of Palestinian patients with COVID-19: A multi-center descriptive** **phenomenological study of recovery journey**

Aidah Alkaissi^1*^, Fadi Zaben^1^, Mohammad Abu-Rajab^1^, Mahdia Alkony^1^

^1^Nursing and Midwifery Department, Faculty of Medicine & Health Sciences- An-Najah National University

^*^Correspondence: Dr. Aidah Alkaissi. Nursing and Midwifery Department, Faculty of Medicine & Health Sciences- An-Najah National University, E.Mail: [aidah@najah.edu](mailto:aidah@najah.edu), Mobile: 00970 (0)597395520

**Supplementary Table S1:** Adherence to COnsolidated criteria for REporting Qualitative research (COREQ) Checklist [[1](#_ENREF_1)]

| **#** | **Topic** | **Guide Questions/Description** | **Page/Line # in the manuscript** |
| --- | --- | --- | --- |
|  | **Domain 1: Research team and reﬂexivity** |  |  |
|  | *Personal characteristics* |  |  |
| 1 | Interviewer/facilitator | Which author/s conducted the interview or focus group? | Provided in the Methods section, under: Data collection |
| 2 | Credentials | What were the researcher’s credentials? E.g. PhD, MD | Provided in the Methods section, under: Data collection |
| 3 | Occupation | What was their occupation at the time of the study? | Provided in the Methods section, under: Data collection |
| 4 | Gender | Was the researcher male or female? | Provided in the Methods section, under: Data collection |
| 5 | Experience and training | What experience or training did the researcher have? | Provided in the Methods section, under: Data collection |
|  | *Relationship with participants* |  |  |
| 6 | Relationship established | Was a relationship established prior to study commencement? | Provided in the Methods section, under: Participants |
| 7 | Participant knowledge of the interviewer | What did the participants know about the researcher? e.g. personal goals, reasons for doing the research | Provided in the Methods section, under: Participants |
| 8 | Interviewer characteristics | What characteristics were reported about the inter viewer/facilitator? e.g. Bias, assumptions, reasons and interests in the research topic | Provided in the Methods section, under: Participants |
|  | **Domain 2: Study design** |  |  |
|  | *Theoretical framework* |  |  |
| 9 | Methodological orientation and Theory | What methodological orientation was stated to underpin the study? e.g. grounded theory, discourse analysis, ethnography, phenomenology, content analysis | Provided in the Methods section, under: Data analysis |
|  | *Participant selection* |  |  |
| 10 | Sampling | How were participants selected? e.g. purposive, convenience, consecutive, snowball | Provided in the Methods section, under: Participants |
| 11 | Method of approach | How were participants approached? e.g. face-to-face, telephone, mail, email | Provided in the Methods section, under: Participants |
| 12 | Sample size | How many participants were in the study? | Provided in the Results section, in the first paragraph |
| 13 | Non-participation | How many people refused to participate or dropped out? Reasons? | N/A |
|  | *Setting* |  |  |
| 14 | Setting of data collection | Where was the data collected? e.g. home, clinic, workplace | Provided in the Methods section, under: Data collection |
| 15 | Presence of non-participants | Was anyone else present besides the participants and researchers? | Provided in the Methods section, under: Data collection |
| 16 | Description of sample | What are the important characteristics of the sample? e.g. demographic data, date | Provided in the Results section and in Table 1 |
|  |  | | |
| 17 | Interview guide | Were questions, prompts, guides provided by the authors? Was it pilot tested? | Provided in the Methods section, under: Data collection |
| 18 | Repeat interviews | Were repeat interviews carried out? If yes, how many? | Provided in the Methods section, under: Data collection |
| 19 | Audio/visual recording | Did the research use audio or visual recording to collect the data? | Provided in the Methods section, under: Data collection |
| 20 | Field notes | Were ﬁeld notes made during and/or after the interview or focus group? | Provided in the Methods section, under: Data collection |
| 21 | Duration | What was the duration of the inter views or focus group? | Provided in the Methods section, under: Participants |
| 22 | Data saturation | Was data saturation discussed? | Provided in the Methods section, under: Data collection |
| 23 | Transcripts returned | Were transcripts returned to participants for comment and/or correction? | Provided in the Methods section, under: Participants |
|  | **Domain 3: analysis and ﬁndings** |  |  |
|  |  | | |
| 24 | Number of data coders | How many data coders coded the data? | Provided in the Methods section, under: Data analysis |
| 25 | Description of the coding tree | Did authors provide a description of the coding tree? | Provided in the Methods section, under: Data analysis |
| 26 | Derivation of themes | Were themes identiﬁed in advance or derived from the data? | Provided in the Methods section, under: Data analysis |
| 27 | Software | What software, if applicable, was used to manage the data? | N/A |
| 28 | Participant checking | Did participants provide feedback on the ﬁndings? | N/A |
|  | *Reporting* |  |  |
| 29 | Quotations presented | Were participant quotations presented to illustrate the themes/ﬁndings? Was each quotation identiﬁed? e.g. participant number | Provided in the results section |
| 30 | Data and ﬁndings consistent | Was there consistency between the data presented and the ﬁndings? | Provided in the Methods section, under: Data analysis |
| 31 | Clarity of major themes | Were major themes clearly presented in the ﬁndings? | Provided in the results section |
| 32 | Clarity of minor themes | Is there a description of diverse cases or discussion of minor themes? | Provided in the results section |

**Supplementary Table S2:** The interview guide

| **Demographic data** | |
| --- | --- |
| Coding Number | |
| Name |  |
| Age |  |
| Gender |  |
| Place of residence | |
| Telephone number | |
| Marital status | |
| Number of offspring | |
| Number of family members | |
| Level of education | |
| Smoking status | |
| Occupation | |
| Comorbidities | |
| Monthly income | |
|  |  |
| **Interview questions** | |
| How did you get the news about your test result when they told you that you tested positive and that you had Coronavirus? | |
| How would you describe how you felt when you were taken to solitary isolation in a place you did not know? | |
| How was the Corona virus transmitted to you? | |
| Did you transmit the virus to another person? | |
| How would you describe your relationship / feeling with the person who infected you? | |
| How your symptoms developed (from the first symptom to recovery) | |
| How did you spend the day during illness? (Isolation and quarantine) | |
| Describe to me the situations that bothered you the most and that you do not want to remember during your infection with the virus? | |
| The situations that affected you the most and changed your life after you got the disease? | |
| What methods did you use to overcome the disease or cope with your situation after the injury? | |
| How was it? Describe how you felt when you received the news of your recovery and left the isolation | |
| What does it mean to you that you were infected with Coronavirus and that you have recovered from the disease? | |
| What advice would you give to people who are worried about Coronavirus? | |
| Do you want to add something that is not mentioned? | |

**Additional quotations on the major themes and subthemes**

### Major theme 1: Emotions after learning about the infection

#### Subtheme 1: Shock/disbelief (feeling awful, anxious, and shocked)

*“…….honestly, I was shocked and felt that this was the end of my life” (P15)*

#### Subtheme 2: Denial (feeling doubtful)

*“I did not complain of any symptoms. Because of my husband's infection I did a PCR and I tested positive. On the third day, signs of the disease began to appear. My body temperature was 39.5 ºC. I had nausea, vomiting, pain in the upper back and legs, and a strong desire to sleep” (P4)*

*“I thought I would never contract it [COVID-19], but eventually I did. I took my precautions. I wore masks, gloves, and I felt confident that I was taking all the needed precautions” (P 1)*

#### Subtheme 3: Feeling angry/frustrated

*“Sometimes I felt like I was in jail” (P1)*

#### Subtheme 4: Bargaining/spirituality to cope with adversity

*“I used to surf the internet to search for information about the disease and news about any treatment. Sometimes, I used to read the Holy Quran” (P9)*

#### Subtheme 5: Feeling guilty

*“I feel guilty because of the potential risks my family were exposed to. I was worried that my family could contract the virus” (P19)*

*“I had anxiety when I thought that my family members could get infected. During my isolation period, I was worried that my family members were left alone” (P1)*

*“I was feeling guilty and embarrassment when I thought that I have passed the infection to someone who came in contact with me” (P2)*

#### Subtheme 6: Depression (feeling depressed and lonely)

*“I could not sleep; I was afraid to die without being able to see my family and friends. Despair overcame me.” (P17)*

#### Subtheme 7: Acceptance and hope (being positive)

*“I was very happy when I learned that I was cured and that the result of COVID-19 was negative. Praise be to God always and forever” (P13)*

### Major theme 2: Experiencing social discrimination and stigma

#### Subtheme 1: Social discrimination and stigma while living with the infection

*“Names of the patients who tested positive for coronavirus used to go viral on social media. I learned about my infection though posts on social media” (P11)*

*“The neighbors burnt my trash can in the street in front of my house when they noticed that the ambulance came to take me to the COVID-19 isolation facility” (P1)*

*"I was being stigmatized by being linked to a confirmed case” (P1)*

*“I faced backlash and harsh comments from the public, even those who knew me” (P4)*

*“People reproached me for contracting the virus. They blamed me for not taking adequate precautions” (P9)*

#### Subtheme 2: Social discrimination and stigma after recovery

*“After recovery, people used to change the road to avoid me when I walked in the street” (P20)*

*“I was most bothered by some of my relative who told me to get away from them and never to come close to them” (P10)*

### Major theme 3: The experienced symptoms

*“I had cold-like symptoms at the beginning before being diagnosed with COVID-19” (P2)*

*“I had a high fever and pain that tortured every part of my body…..I kept on coughing specially in the afternoon. I could not sleep at all…The symptoms lasted for two days” (P20)*

*“The last few days were awful. I lost the senses of smell and taste. I had a high temperature of around 39 ºC, cough, sore throat, breathlessness, nausea, and vomiting” (P14)*

*I was utterly exhausted. I had whole-body ache and felt weak. I was feeling tired” (P4)*

*“I felt tired… literally like a rag. I could not stay awake for long; I have to go to bed” (P13)*

*“I felt pain in the upper part of my back, especially the shoulder areas. I felt that my shoulders were flared” (P4)*

*“I had productive cough with brown purulent mucous” (P2)*

*“I had headache that started in the upper frontal part of my head” (P4)*

*“I decided to go for a PCR test after experiencing a very dry throat. My throat used to burn, especially in the night. This was the worst sore throat I ever experienced. I felt thirsty and water did not quench my thirst” (P19)*

*“…. People who saw me asked me why my eyes were red” (P5)*

*“I did not feel anything because shortness of breath has taken over my feelings. I felt that I would suffocate to death” (P13)*

*“I suffered stomachache, diarrhea, dizziness, and I had blurred eyes” (P15)*

### Major theme 4: Supportive treatments, herbs, rituals, and social support

#### Subtheme 1: Supportive treatments and herbs

*“Fruits and juices are important to build immunity” (P5)*

*“I was eating healthy food, consuming a lot of water, and using various types of natural herbs” (P5)*

#### Subtheme 2: Rituals

*“I used to stay in prone position. This position was good to relief cough, especially, in the night” (P1, P4)*

#### Subtheme 3: Social support

*“My wife used to bring me various food and drinks, follow up with my treatment, and take care of me in all aspects of life” (P2)*

*“I was always in touch with my family and my wife. This assured me and helped me pass this difficult isolation period” (P11)*

*“This disease made me realize that my loved ones were many. I have seen my beloved ones standing by my side all the time. Staying in contact with them helped relief my pains and weaknesses. They were part of the treatment and improved my spirits” (P6).*

*“My wife and I were tested positive for COVID-19. We stayed in isolation for 21 days. During this period, I was relieved the pressure of work and organized my life. I came out of isolation in a good mood. Being imprisoned with my beloved one was sweet” (P10)*

### Major theme 5: Life after recovery

#### Subtheme 1: Feeling relieved

*“When I got out of isolation after 23 days, I felt that I am free and as if I was in a prison” (P6)*

*“I feel released! ... As if I was in prison and I was pardoned .... I swear to God, it was good news to be released” (P16)*

#### Subtheme 2: Adherence to preventive measures and adopting a healthy lifestyle

*“The use of masks, sanitizers, frequent hand washing, and practicing physical distancing would help prevent the disease” (P4)*

#### Subtheme 3: Fear of re-infection

*“I am not happy because the symptoms were not completely over yet. I feel that I could relapse and return to the same suffering at any time. I felt tired earlier today, even though it has been more than 25 days after my recovery from the disease” (P6)*

*“I pray to God that the disease will not return back to me. For the first time in my life, I experienced such suffering. I did not expect to return to my normal life” (P6)*
